# Universal Real-Time PCR-Based Assay for Lentiviral Titration

**DOI:** 10.1007/s12033-014-9815-4

**Published:** 2014-11-05

**Authors:** Wojciech Barczak, Wiktoria Suchorska, Błażej Rubiś, Katarzyna Kulcenty

**Affiliations:** 1 Radiobiology Lab, Department of Medical Physics, The Greater Poland Cancer Centre, Garbary 15 Str., 61-866 Poznan, Poland; 2 Department of Head and Neck Surgery, The Greater Poland Cancer Centre, Poznan University of Medical Sciences, Poznan, Poland; 3 Department of Clinical Chemistry and Molecular Diagnostics, Poznan University of Medical Sciences, Przybyszewskiego 49, 60-355 Poznan, Poland; 4 Department of Cancer Immunology, Chair of Medical Biotechnology, Poznan University of Medical Sciences, Poznan, Poland; 5 Gene Therapy Laboratory, Department of Cancer Immunology, Greater Poland Cancer Centre, Poznan, Poland

**Keywords:** Lentivirus, qPCR, Titration methods, WPRE, Albumin

## Abstract

Lentiviral vectors are efficient vehicles for stable gene transfer in both dividing and non-dividing cells. This feature among others makes lentiviral vectors a powerful tool in molecular research. However, the use of lentiviruses in research studies and clinical trials requires a precise and validated titration method. In this study, we describe a qPCR-based approach for estimation of lentiviral vector titer (pLV-THM-GFP). The use of WPRE (Woodchuck Hepatitis Virus Posttranscriptional Regulatory Element) and albumin genes as templates for an SYBR green-based real-time qPCR method allows for a rapid, sensitive, reproducible, and accurate assessment of lentiviral copy number at an integrated lentiviral DNA level. Furthermore, this optimization enables measurement of lentiviral concentration even in very poor quality and small quantity material. Consequently, this approach provides researchers with a tool to perform low-cost assessment with highly repeatable results.

## Introduction

Lentiviral vectors are efficient vehicles for stable gene transfer in dividing cells, as well as in non-dividing cells such as neurons or hepatocytes [1, 2]. Production of lentiviral vectors is achieved by transfection of HEK293T cells using high concentrations of different plasmids. Transduction by lentiviral vectors matches a single-round infection and results in constant integration into the genome of both dividing and non-dividing cells. However, in order to apply lentiviral methods in research and clinical studies, it first requires assessment of titers.

To date, various methods of functional and non-functional lentiviral titration have been described, including p24 antigen ELISA (enzyme-linked immunosorbent assay), RNA titers, reverse transcriptase (RT) activity, dot blot, fluorescence-activated cell sorting (FACS), and quantitative polymerase chain reaction modifications (qPCR) [3, 4]. However, most of these methods overestimate the functional titer. For instance, the p24 protein pool includes a variable amount of free p24 and p24 associated with non-functional vector particles [5]. Non-functional particles are also measured by the RNA titration method. RT-assay, which is a quite rapid method, presents disadvantages in measuring RT activity in both functional and non-functional vectors [6], mostly due to the multiple stages involved in the process that lead to increased pipetting errors and decreased reproducibility. A more accurate method of titrating functional vectors is via cell transduction with lentiviral vector dilutions. Other methods in use include evaluation of reporter protein activity and assessment of the number of colony forming units.

The most precise approaches to quantifying the titer of functional vectors are based on FACS and qPCR analysis. While FACS analysis gives an equal quantification of viral titer, it is limited to the vectors with expression of fluorescent transgenes (e.g., green fluorescent protein [GFP] or specific antibodies), and cannot discriminate cells with single or multiple integrants [7]. A more advanced FACS-based method can increase titration accuracy has been described, although that assay can take up to 52 h (12–52 h), depending on the virus [8].

Generally, the titer of a functional vector is defined as the number of vector particles required to transduce a single cell in a defined volume. In that case, the most accurate measurement of the number of functional particles can be obtained by estimating the number of integrated DNA lentiviral copies per cell by a universal, real-time PCR assay [9, 10]. Modified RT-qPCR methods to quantify lentiviral mRNA copies after transduction in cell culture have also been described [11, 12]. Without fluorescent genes, it is necessary to estimate expression at the mRNA or DNA level by measuring the number of copies of stably integrated lentiviruses. Therefore, real-time PCR methods allow for rapid and precise quantitation of lentiviral transgene mRNA expression in transduced target cells. Furthermore, use of the Woodchuck Hepatitis Virus Posttranscriptional Regulatory Element (WPRE) sequence, which is often built into lentiviral vectors, makes the method widely applicable.

In this paper, we described a method, based on quantitative PCR (qPCR) assay, to measure the number of copies of lentiviruses stably integrated into the genome after production and transduction. The qPCR method for lentiviral titration analysis described here is not necessarily novel [9, 13–16]. However, we have optimized the technique to produce accurate, reproducible results in a cost-efficient assay.

## Materials and Methods

### Lentiviral Vector

For these studies, we utilized the pLV-THM-GFP lentiviral vector which expresses the GFP gene under control of the H1 promoter (Plasmid #12247; Addgene; Cambridge [USA]).

### Cell Culture

To assess the lentiviral titer, the HEK293T (ATCC number: CRL-3216) cell line was cultured in standard conditions. Briefly, cells (50 % confluence, max. 3rd passage) were cultured in DMEM medium supplemented with 10 % fetal bovine serum (Sigma-Aldrich) and 1 % penicillin/streptomycin (Sigma-Aldrich). Experiments were performed in 24-well plates (50,000 cells per well) for 96 h. After media changing, lentiviruses obtained according to a previously published protocol [17] were added to wells in different volumes (4 or 2 µl). Transduction was performed in the presence of polybrene (5 µg/ml, Sigma-Aldrich). After 96 h incubation (reducing the contamination from plasmid DNA) at 37 °C and 5 % CO_2_, cells were harvested and DNA was isolated.

### DNA Isolation

DNA was extracted from HEK297T cells using a mini column-based DNA isolation kit (A&A Biotechnology; Gdynia, Poland) and stored at −20 °C, according to manufacturer’s protocol as previously described [18]. High concentration standard samples of plasmid and oligonucleotide DNA were prepared in decimal concentrations to cover all possible measurement ranges.

### Real-time PCR

The concentration of lentivirus was assessed with two pairs of primers: a lentiviral-specific transgene (WPRE gene) and a single copy gene-specific reference gene (albumin), according to standard procedure. The primers (Table 1) were tested in the context of specificity. A detailed analysis and optimization of the reaction protocol were performed to obtain a precise description of the efficiency of individual reactions. The protocol was optimized to eliminate primer-dimer formation even after 45 cycles of amplification or in low DNA content samples. These steps enabled validation of the method and, consequently, more reliable and reproducible results. Additionally, the protocol was performed on a base of the universal SYBR green dye in a mono-color reaction, which makes the method easy to implement in any qPCR platform.

**Table 1:** Primer sequence used for titration assay in order to amplify Albumin and WPRE fragments

Name	Primer sequence
ALB F	5′-TTTGCAGATGTCAGTGAAAGAGA-3′
ALB R	5′-TGGGGAGGCTATAGAAAATAAGG-3′
WPRE F	5′-GTCCTTTCCATGGCTGCTC-3′
WPRE R	5′-CCGAAGGGACGTAGCAGA-3′

**Fig. 1:**
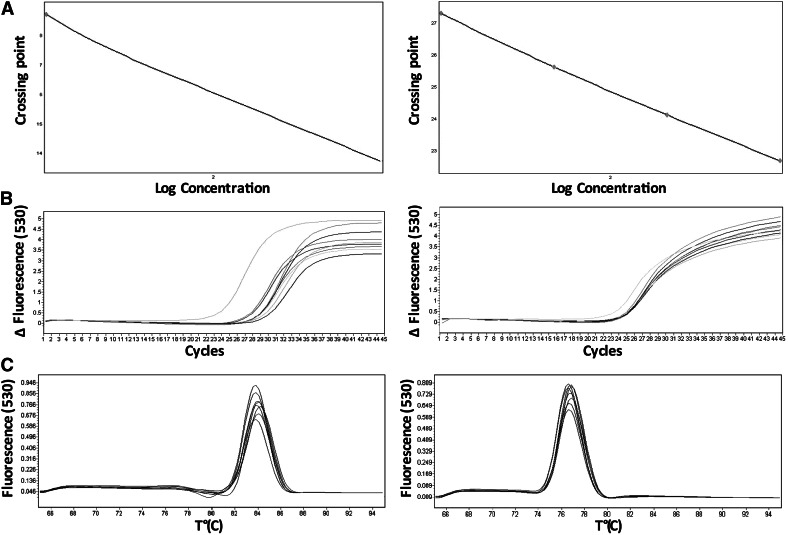
Quantitative and qualitative assessment of WPRE fragment relative to single copy gene, albumin. **a** standard curve for gene copies for albumin (*right*) and WPRE (*left*). Crossing point—a cycle in which the fluorescence level rises above the baseline; **b** typical quantitative PCR analysis of reference gene (*right*) and WPRE (*left*); **c** typical qualitative qPCR analysis based on melting temperature of amplicons: WPRE (*left*) and single copy gene, albumin (*right*)

Next, the pair of primers specific to the single copy gene (albumin) (Table 1) was designed, and a new protocol for both amplicons that reveals specific reaction and efficiency close to 100 % was proposed. The primer concentration and the annealing temperatures analysis were also optimized, thus increasing reaction efficiency with simultaneous elimination, a primer-dimer formation. The optimal PCR conditions to amplify the lentiviral-specific fragment (WPRE) and albumin gene (Alb)—both in one run—were carried out as follows: initial denaturation and polymerase activation (hot start) were performed at 95 °C for 5 min without fluorescence acquisition. The signal was detected during another 45 cycles (95 °C/10 s, 60 °C/10 s, and 72 °C/10 s). Melting analysis (65–95 °C range, 0.11 °C resolution) at the end of the reaction was performed to verify specificity of the product; that analysis indicated *Tm* = 84.5 (WPRE gene) and *Tm* = 77.2 (albumin). The efficiency of the reaction was no lower than 94.4 %. Importantly, this result was repeatable for all samples that were analyzed (in serial dilutions). After optimization, the optimal concentration of primers was estimated at 1 µM. Standard curves for WPRE and albumin genes were obtained using plasmid carrying WPRE gene and designed oligonucleotides (albumin) (Fig. 1). The lentiviral titer was assessed using a qPCR system (LC 480; Roche Diagnostics, Basel, Switzerland) and SYBR Green Master Mix kit (Roche).

### Protocol

1. Day 1—Seed a cell on a plate. Transducing HEK293T cells with the lentiviral vector of interest in 24-well tissue culture plates.
2. Day 2—Change fresh medium. Incubation for 96 h to reduce contamination from plasmids DNA possibly transfected with the vector.
3. Day 5—Harvest cells and extract cell DNA from each individual well of the 24-well plate.
4. Day 5—Perform Absolute Quantification qPCR reaction.

### Functional DNA Titer of pLV-THM-GFP Vector by FACS

**Fig. 2:**
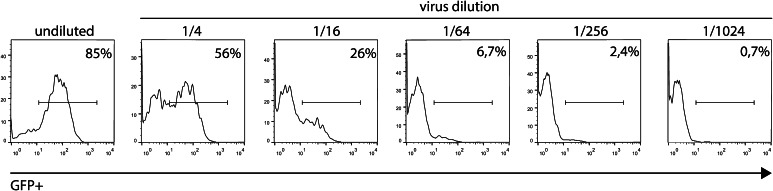
Flow cytometry estimated for the lentiviral titer of virus carrying GFP cassette; Histograms showing GFP-positive cells, 72 h after transduction with 4-fold dilutions of lentiviral vector

For FACS lentiviral titration, we used lentivirus carrying GFP because its fluorescence can be easily measured using flow cytometry analysis. The experiment was performed on HEK293T cells in a 24-well plate (20,000 cells per well). At 24 h post-seeding, cells from the one well were counted (to determine the number of transducing cells) and transduced with serial 4-fold dilutions of lentiviral: undiluted 1/4, 1/16, 1/64, 1/256, and 1/1024. After 72 h, cells were detached from the plate (using trypsin–EDTA) and the lentiviral biological titer was estimated on FACS based on GFP fluorescence. (Figure 2)

## Results

### qPCR

The studies revealed that lentiviral titer can be easily assessed with the use of qPCR based on the SYBR Green-based detection system. Moreover, the method was rapid and reproducible.

The number of lentiviral vector copies was calculated by Absolute Quantification with LightCycler 480 2.0 Software based on standard curves for transgene (WPRE) and estimation of absolute DNA titers was achieved by comparing crossing point values derived from DNA samples to those obtained from a standard curve of known concentrations of plasmid lentiviral DNA (4.5 × 10^7^ to 4.5 × 10^4^ copies/reaction). For albumin, a standard curve was also prepared (6 × 10^7^ to 7 × 10^4^ copies/reaction). The number of copies/µl was calculated according to the following formula:

Number of copies = (*X* ng × 6.0221 × 10^23^ molecules/mole)/[(*N* × 660 g/mole) × 1 × 10^9^ ng/g],

where *X* is the amount of amplicon (ng), *N* is the Length of dsDNA amplicon, and 660 g/mole is an average mass of 1 bp dsDNA

Lentiviral titer (TU/mL; viral particles per mL of supernatant able to transduce target cells) (Table 2) was determined by considering the following parameters according to protocol: primary number of cells seeded in Day 1, lentiviral copy number per cell, and volume of used lentivirus according to the formula given below.

**Table 2:** Estimation of lentiviral titer and the calculation schema for exemplary lentivirus

Volume (µl)	Alb (number of copies)	WPRE (number of copies)
4	302,000	15,000
4	321,000	14,500
2	301,000	11,900
2	340,000	11,400

Volume (µl)	Alb average	WPRE average
4	311,500	14,750
2	320,500	11,650

Volume (µl)	Lentiviral copy number per cell in the sample
4	0.094703
2	0.072699

Volume (µl)	Titer (TU/ml, Transducing Unit)
4	1.2 x 10^6^ ± 7.9 x 10^4^
2	1.8 x 10^6^ ± 2.1 x 10^5^

Lentiviral titer (TU/mL; viral particles per mL of supernatant able to transduce target cells) was determined by considering the following parameters according to protocol: by taking primary number of cells, lentiviral copy number per cell, and volume of used lentivirus. Titration was performed in dual repetitions which gives more accurate and shows repeatable results. The average for single copy gene and WPRE sequence was estimated followed by evaluation of lentivirus titer with formula: lentiviral copy number per cell = (copy number WPRE/copy number Alb) × 2. The 2-fold factor reflects the presence of two alleles of albumin gene. Titer (TU/ml) = (Primary number of cells [50,000 cells] count in day 1 × lentiviral copy number per cell of the sample)/volume of used lentivirus (ml)

*WPRE* Woodchuck Hepatitis Virus Posttranscriptional Regulatory Element; *Alb* albumin (single copy gene)

Calculation Schema:

Lentiviral copy number per cell = (copy number WPRE/copy number Alb) × 2,

Titer (TU/ml) = (Primary number of cells count in day 1 × lentiviral copy number per cell of the sample)/volume of used lentivirus (ml), and

qPCR titer = 1.8 × 10^6^ ± 2, 1 × 10^5^

When the analysis was performed with these standards, it showed that the protocol was high efficacious and specificity. Efficacy was no lower than 94.4 % and there were no observed primer-dimer fragments during the melting curve analysis in either the WPRE or albumin amplicons. As we have demonstrated, the value of lentiviral copy number per a single cell calculated by normalizing the WPRE copy number against the albumin gene copy in the same run might be a very useful approach.

### FACS

For the calculation of the lentiviral titer, we used the GFP-positive value that was closest to 4-fold dilution.

Calculation Schema:

Titer (TU/ml) = (number of transduced cells in day 1 × percent of GFP-positive cells × 1,000/volume of lentivirus used (ul).

FACS titer = 30,000 × 0.067 × 1,000/0.07 = 2.8 × 10^7^.

## Discussion

Numerous methods have been described for lentiviral titration, including quantitation of viral particles in vector supernatants, measuring integrated proviral DNA in target cells, and quantification of transgene-encoded protein in target cells. The novel SYBR green-based real-time qPCR method reported here, based on WPRE and albumin templates, provides a cost-effective, accurate, and reproducible method of lentiviral titration.

The most useful assessment of functional viral titration is those methods that permit detection of either transgene mRNA or protein expression levels. Titration methods based on protein expression require a fluorescent gene reporter (GFP, luciferase), which is not available in all lentiviral vectors, or staining with an antibody against the transgene. Target cell expression of transgene-encoded fluorescent or luminescent protein products can also be easily detected by immunofluorescence staining or FACS analyses with specific antibodies against protein reporters.

A few reports (Geraerts et al., Lizee et al.) have described the application of the qPCR method for lentiviral titration [4, 12]. Those studies describe a quantitative RT-PCR method using primer sequences specific for the WPRE, which gives functional transgene expression of lentiviral particles. However, titration on the mRNA level may yield results that differ from those obtained by estimating the number of lentivirus copies on the DNA level. Studies have demonstrated that integration events do not necessarily correlate with viral gene expression, because a significant proportion of provirus integrates into regions of the genome that are not amenable to gene transcription. Differences between proviral DNA and expression level could depend on promoter force or specification of transduced cells. More importantly, both proviral DNA and expression measurement provide a more accurate titer than RNA titration (even 1,000-fold lower titer) [9]. Furthermore, those methods are based on two pairs of fluorescent probes defined for specific systems (Applied Biosystems), which increases cost of analysis.

In 2009, Kutner et al. described an approach to lentivirus titration based on qPCR with fluorescent probes [19]. Our results show that, analogous to the use of probes, an optimized application of SYBR green-based method for all real-time PCR systems is available in laboratories. This optimization and usage of real-time PCR equipment enable measurement of lentiviral concentration even in very poor quality materials in low quantities (even with just one copy of the lentivirus). We were able to achieve close to 100 % efficiency which means that it is possible to detect a single copy of the sample using most qPCR platforms. The advantage of this method is the identification of optimal conditions for individual amplicons with a lack of competitive amplification that might affect the reaction efficiency. When providing consistent conditions, we were able to increase the number of cycles up to 45 with no significant presence of primer-dimer structures. This modified technique provides a fast method of quantifying the lentiviral concentration. Other important advantages of this approach are its simplicity, the use of common reagents available in most molecular biology laboratories, and the ability to measure functional particles. All of these advantages enable the use of this approach in both research and clinical studies. Moreover, with this technique, lentiviral vectors could also be used in interdisciplinary clinical research, for example, in radiotherapy [20].

## Conclusions

In conclusion, the use of WPRE and albumin as templates for real-time qPCR based on SYBR green staining allows for a sensitive and accurate assessment of lentiviral copy numbers at integrated lentiviral DNA level. Lentivirus titering analysis can be widely applicable, in both research and clinical studies and, importantly, it does not depend on reporter gene expression analysis.
